# A unique approach to faculty development using an Observed Structured Teaching Encounter (OSTE)

**DOI:** 10.1080/10872981.2018.1527627

**Published:** 2018-10-21

**Authors:** Miriam A Smith, Regine Cherazard, Alice Fornari, Patti Adelman, Michelle Snopkowski, Martin Lesser

**Affiliations:** a Department of Medicine, Long Island Jewish Forest Hills, Donald and Barbara Zucker School of Medicine at Hofstra/Northwell Health System, Forest Hills, NY, USA; b Faculty Development, Donald and Barbara Zucker School of Medicine at Hofstra/Northwell Health System, Hempstead, NY, USA; c Center for Learning and Innovation, Physician Leadership Institute Northwell Health System, Hempstead, NY, USA; d Learning and Organizational Development Specialist, Center for Learning and Innovation and Physician Leadership Institute Northwell Health System, Hempstead, NY, USA; e Department of Molecular Medicine and Population Health, Feinstein Institute for Medical Research Northwell Health System, Hempstead, NY, USA

**Keywords:** Observed Structured Teaching Encounter (OSTE), faculty development, teaching and feedback skills

## Abstract

We have challenges with poor patient satisfaction scores (Hospital Consumer Assessment of Healthcare Providers and Systems [HCAHPS]) and internal medicine resident (IMR) evaluations of voluntary attending physicians. Using an Observed Structured Teaching Encounter (OSTE), we designed a faculty development project that focused on attendings’ teaching and feedback skills. To assess attending communication with interns and improve attending teaching and feedback skills. All IM attendings on the Long Island Jewish Forest Hills (LIJFH) Emergency Department (ED) call schedule participated. OSTE simulation sessions included two clinical scenarios, standardized patients (SPs), fourth-year medical students trained as ‘interns,’ OSTE checklists, and debriefing. We analyzed ‘intern’ ratings of communication with attendings and attending self-assessment during the OSTE, and attending HCAHPS scores and IMR evaluations of attendings pre- and post-OSTE. Twenty-nine of 29 attendings completed the OSTE. Although an increase was demonstrated pre- to post- for ‘intern’ OSTE ratings of attendings and LIJFH attending self-assessment ratings, there was no statistically significant difference. Mean HCAHPS scores and resident evaluations of attendings also increased from pre- (22% and 3.59) to post-OSTE (30% and 3.87) but did not reach statistical significance. A statistically significant difference for both cases was demonstrated when comparing mean attending self-assessment ratings with ‘intern’ evaluation of attendings. Attending teaching/feedback skills improved between cases, attending self-ratings were higher than ‘intern’ ratings of attendings. HCAHPS and IMR evaluations of attendings improved post-OSTE. Regular intervention utilizing an OSTE may provide a sustained benefit for enhancing attendings’ skills, patient satisfaction, and resident training.

## Introduction

Long Island Jewish Forest Hills (LIJFH), a major affiliate of a large healthcare system (Northwell Health System), is a 312-bed, academic, community hospital in the NY metropolitan area with an ethnically diverse patient population and a training program of 38 internal medicine residents (IMR). Approximately 94% of hospitalized patients are admitted through the Emergency Department (ED) which handles about 57,000 visits annually. Although our hospitalist service has been growing, at the time of the study, 75% of admitted, ‘unattached’ patients were admitted to IMR and voluntary attending physicians; 25% to IMR and full-time hospitalists.

We have ongoing challenges with poor patient satisfaction scores (Hospital Consumer Assessment of Healthcare Providers and Systems [HCAHPS]) [1]. For the past 4 years, HCAHPS rating of communication for all voluntary LIJFH Department of Medicine staff physicians who admit patients to the hospital has not exceeded the 7th percentile. In addition, resident evaluations of voluntary faculty at LIJFH have been below average.

Methods to address faculty development in the context of competency-based medical education have been implemented and evaluated. An Observed Structured Teaching Encounter (OSTE) initially described in 1992 uses a standardized encounter to assess performance and enhance teaching skills [2]. We designed an OSTE simulation study using a novel approach to faculty development. We included standardized patients (SPs), standardized ‘interns’ (fourth-year medical students [MS4s] from the Donald and Barbara Zucker School of Medicine at Hofstra/Northwell [‘evaluators’]), and general medicine attending physicians (the ‘learners’) on the LIJFH ED call schedule to assess attending communication with house staff and improve attending teaching and feedback skills. We were also interested in evaluating the extent to which an improvement in attending physicians’ competencies during a simulation exercise could be demonstrated in an authentic patient care and resident training environment.

## Methods

This faculty development project was submitted and approved through expedited review by the Northwell Health Institutional Review Board (New Hyde Park, NY). The study took place from September 2015 through February 2017.

Multiple focus groups were conducted in the winter 2015 by personnel from the Northwell Center for Learning and Innovation (CLI) with IM LIJFH voluntary and full-time hospitalist attending staff on the general medicine ED call schedule, and the LIJFH IMR to determine barriers to communication and teaching. OSTEs were conducted at CLI (simulation center).

Many voluntary faculty in medicine admit patients to LIJFH. However, only 29 attending physicians are on the general medicine ED call schedule to admit ‘unattached’ patients and all 29 physicians were required to participate in the study. No financial incentive was provided to the faculty participants. Twenty-six of 29 attendings were voluntary faculty from the community; 3 were full-time hospitalists. Twelve MS4s served as ‘interns’ and were given a small stipend to participate. SPs were trained for each clinical vignette with one SP in the room for each case. Four Zucker School of Medicine faculty members trained in Self-Assessment-Feedback-Encouragement-Direction (SFED) observed the exercise and facilitated a group debriefing [3].

In March 2016, separate teaching sessions to train MS4s as standardized ‘interns’ and the SPs in their role as patients were held at CLI. The MS4s were trained over 2–3 h by medical school faculty expert in OSTE simulation. They were given two common clinical scenarios which incorporated detailed information about the patient’s medical history, physical examination findings, and laboratory/imaging results and instructed on the learning objectives and evaluation tools. They also received clear instructions regarding responses to potential questions the faculty member may ask and, more specifically, how much information the MS4 could disclose [4].

Three faculty development, OSTE simulation sessions lasting about 2.5 h each were conducted at CLI in April and May, 2016. Each session included a maximum of 10 faculty members. Two clinical case vignettes (hypoglycemia and gastrointestinal bleed) were developed for each OSTE session to simulate a bedside interaction between the ‘intern’ and attending. The case information provided was tailored to the MS4 and attending. More specifically, certain pieces of information were intentionally omitted from the attending’s bedside chart to provide teaching opportunities for the attending. Physical examinations were not performed for either case.

The 29 LIJFH attendings functioned as the ‘learners.’ They were given pre-encounter instructions to proceed with each simulation exercise in a fashion similar to their day-to-day bedside interactions with house staff and patients. There was no special order as to which case was first or second in order to prevent bias. Based on logistics and scheduling issues, we could not fully randomize the MS4s in all OSTE sessions.

**Figure 1.**
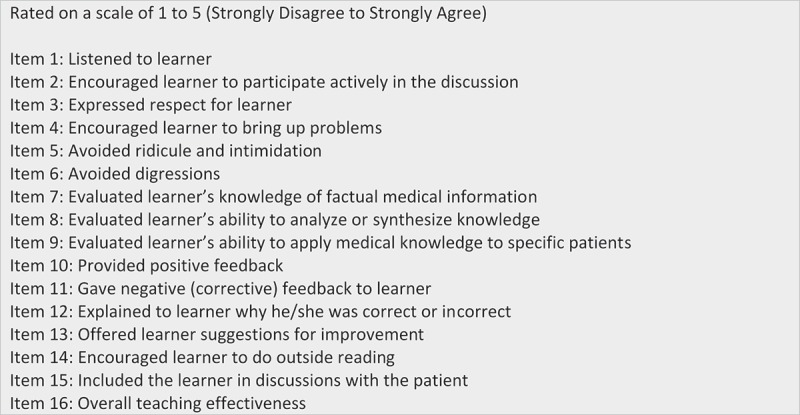
Objective Structured Teaching Encounter (OSTE) questionnaire. Adapted from Morrison EH, Boker JR, Hollingshead J et al. [5].

Post-encounter, validated OSTE checklists for ‘intern’ evaluation of the attending and attending self-assessment were used with each case [5] The OSTE checklists were modified to accommodate the clinical scenarios and incorporated a 16-item questionnaire for each case (Figure 1). Group intervention through SFED took place between cases and a debriefing took place at the end of the OSTE. Each clinical case scenario lasted 20 min and consisted of 2 min for case review (i.e., bedside chart) by the” intern” and attending, 3 min for the ‘intern’ case presentation to the attending with the SP present, 10 min for the attending to ‘teach’, and 5 min for checklist completion (‘intern’ evaluation of the attending and attending self-assessment. Each attending received individual OSTE checklist results (self-assessment and ‘intern’ assessments) immediately after conclusion of the exercise. Each encounter was videotaped and available to attendings for self-improvement and individual coaching upon request.

The SFED between cases focused on expectations of an attending physician as a skilled teacher to establish better communication with the intern. The debriefing at the end of the OSTE reemphasized the expectations of a skilled teacher and included differences between encounters. The post-OSTE debriefing also served as a platform to discuss implementation of improved skills, what ‘worked’ during the second patient encounter, and whether the attending physicians felt comfortable with these enhanced skills in order to apply them to the real clinical setting.

We analyzed (1) ‘ intern’ rating of communication with the attending at the time of the OSTE, (2) attending self-assessment during the OSTE, (3) LIJFH attending HCAHPS scores pre- and post-OSTE, and (4) LIJFH IMR evaluations (New Innovations software) of LIJFH attendings pre- and post- OSTE. Press Ganey HCAHPS scores specifically focused on the global domain of ‘communication with doctors’ were collected for all attendings on the LIJFH ED call schedule.

In order to reduce selection bias and address variability in the data, a Paired Wilcoxon Signed-Rank Test was used to test for statistical significance. MS4s (‘interns’) had no prior contact with the general IM attending staff from LIJFH [4].

The HCAHPS scores for LIJFH medicine attendings were collected within 3 months before and 3 months after the OSTE. Due to the timing of the OSTE simulation sessions, pre-test LIJFH resident evaluations of attendings were obtained within 3 months before the OSTE and included current PGY1s, PGY2s, and PGY3s. The posttest resident evaluations of attendings were collected 6 months after the OSTE and excluded current PGY1s who had not participated in the OSTE and PGY3s who graduated from the training program.

## Results

All 29 attending physicians on the general medicine ED call schedule attended the focus groups. The focus group discussions revealed two recurring themes that were described as barriers to communication and teaching: voluntary attendings did not view their roles as teachers and attending physicians complained of some difficulty getting in touch with house staff caring for their patients.

**Figure 2.**
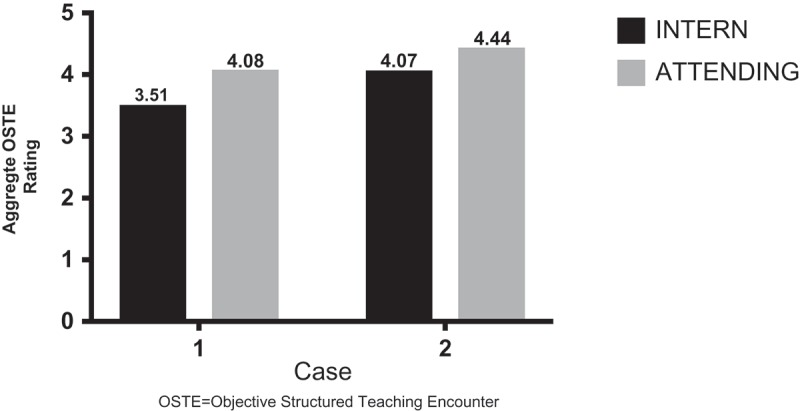
Attending overall self ratings vs. intern ratings. OSTE: Objective Structured Teaching Encounter.

MS4 ‘interns’ completed OSTE checklist ratings of all attendings participating in the OSTE. When comparing overall scores, 76% showed improvement in communication and teaching skills from pre- to post-OSTE. ‘Intern’ mean ratings of attendings increased from case 1 to 2, although this was not statistically significant (Figure 2).

All 29 LIJFH attending physicians participated in the CLI OSTE exercise (100% response rate); 27 completed the self-assessment rating OSTE checklist for both cases. Seventy percent of participants had an overall increase in aggregate ratings from case 1 to 2. The attending self-ratings and the MS4 ‘intern’ ratings demonstrated strong correlation in both cases (0.92 and 0.88, respectively). Attending self-rating aggregate scores increased from case 1 to 2, although not statistically significant (Figure 2). However, a statistically significant difference for both cases was demonstrated when comparing mean attending self-assessment rating scores with ‘intern’ evaluation of attendings (*p* = 0.003 and *p* = 0.01, respectively).

**Figure 3.**
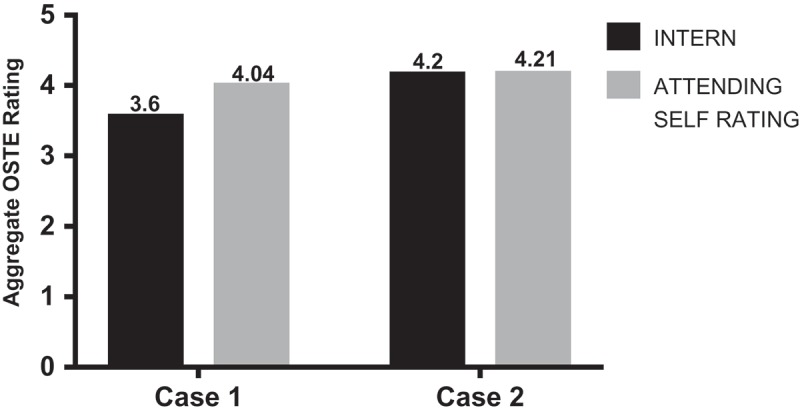
Overall teaching effectiveness.

When isolating the measurement of overall teaching effectiveness (last question on the 16-item OSTE checklist), the mean attending self-rating was higher than the ‘intern’ rating for case 1 (3.6 vs. 4.04). However, both attending self-rating and ‘intern’ mean ratings were higher and almost equivalent for case 2 (4.2 vs. 4.21) (Figure 3).

**Figure 4.**
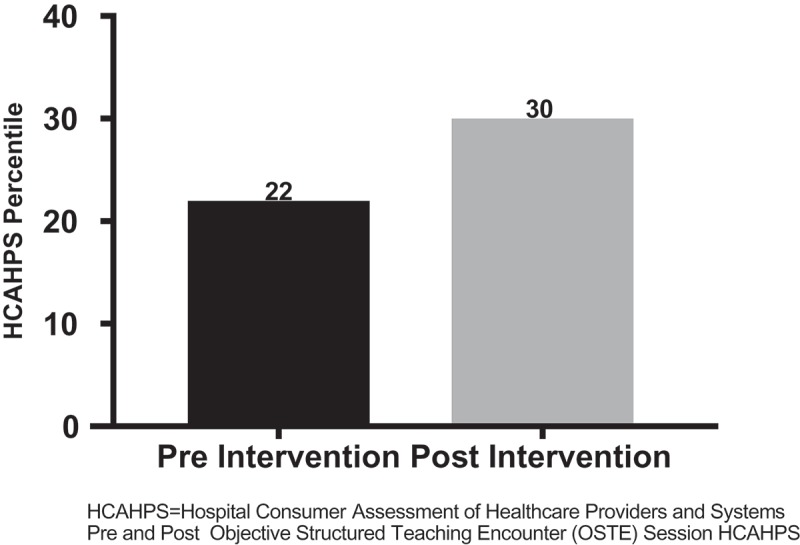
HCAHPS percentile. HCAHPS: Hospital Consumer Assessment of Healthcare Providers and SystemsPre and Post Objective Structured Teaching Encounter (OSTE) Session HCAHPS.

**Figure 5.**
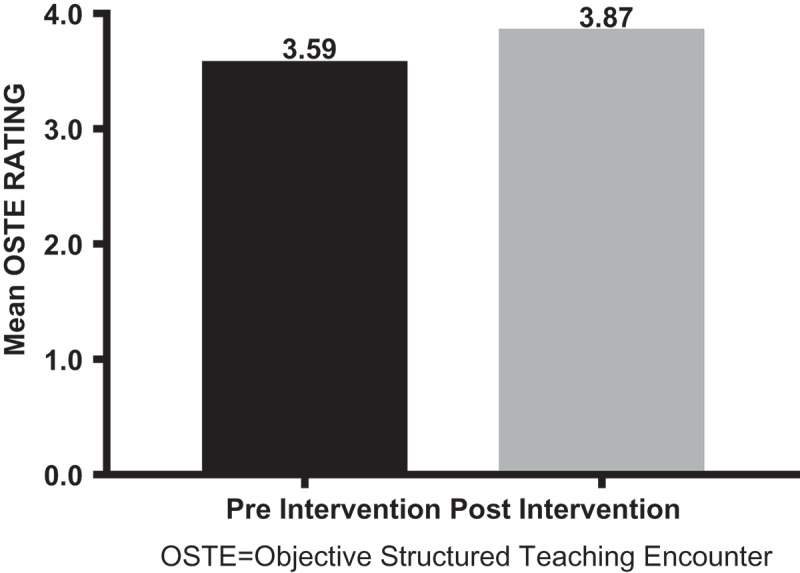
Resident mean evaluation of attendings. OSTE: Objective Structured Teaching Encounter.

Twenty-nine LIJFH attendings had pre-intervention and 21 had post-intervention IMR evaluations. Nineteen attendings had pre-intervention whereas only 16 attendings had post-intervention HCAHPS scores. For the study, HCAHPS results reflect the aggregate means. No ‘weighting’ of HCAHPS results per physician could be done, as those data were unavailable to the investigators. The drop off of post-intervention data was due to attending attrition from LIJFH and/or a voluntary reduction in clinical activity. We did, however, observe an increase in both the aggregate mean HCAHPS scores and IMR evaluations for LIJFH attendings (Figures 4 and 5).

On the attending self-assessment rating questionnaire, ‘listened to the listener’ had the highest average rating for both cases (4.59 and 4.78, respectively). The highest average ‘intern’ rating of attendings was ‘avoided digression’ for case 1 (4.6) and ‘avoided ridicule and intimidation’ for case 2 (4.72). For the attending self-assessment rating and ‘intern’ rating of attendings, ‘encouraged the learner to do outside reading’ had the lowest average rating for both cases (2.92 and 1.48 for case1; 3.4 and 2.38 for case 2).

Attendings were also asked to rate their OSTE experience. The 25 attendings who completed the questionnaire rated the experience as useful to improving their resident teaching and feedback skills (data not shown). Attendings also agreed that the SFED model was a useful tool for strengthening communication skills with residents. Whereas most faculty members commented about their renewed awareness of their roles as educators, a small percentage questioned the value of this exercise. No attending requested to view his/her videotape of the session.

## Conclusions

Efforts to enhance faculty development and to measure teaching and feedback effectiveness are ongoing. OSTEs incorporate methodology to ensure that active learning is occurring and specific teaching competencies can be addressed [6]. The literature supports the impact and reliability of OSTEs as a tool for evaluating teaching skills of medical school faculty, residents, and medical students and seems to support the validity of OSTEs. However, the ideal structure for an OSTE is not as well-defined [7–9]. We designed an OSTE with a unique structure utilizing medical students as ‘intern’ evaluators and attending physicians as learners. We measured pre- and post-OSTE intervention ‘intern’ evaluations of attendings, attending self-evaluations, HCAHPS scores, and IMR evaluations of attending physicians.

There is good evidence that OSTEs serve as an effective tool to assess the benefits of faculty development projects that address various core competencies [4,10,11]. Our OSTE was developed in response to poor attending HCAHPS scores and lower than average resident evaluations of attendings.

The LIJFH attending physician participants were given pre-encounter instructions to proceed with each simulation exercise in a fashion similar to their day-to-day bedside interactions with house staff and patients. A faculty development intervention by medical educators trained in the SFED model of feedback occurred between cases with a debriefing following the standardized simulation sessions at the end of the OSTE. Although no statistical significance was achieved, the brief intervention demonstrated a positive trend with respect to faculty performance when comparing case 1 with case 2. Debriefing after the second case revealed overall satisfaction by the attending with their learning experience and better understanding of their role in communication with ‘interns’ during bedside teaching when patients are present.

There are limitations to this study. The sample size for attending participation and assessment of attending skills was small. Several voluntary attending physicians either left or reduced their clinical time at our institution, which likely had an impact on failure to achieve statistical significance for most measurements. In addition, the faculty development intervention on feedback skills was brief between cases, which may have been insufficient to demonstrate an even greater impact. Finally, the simulation sessions used only one case pre-intervention and one case post-intervention. Multiple cases could have improved data results.

This was a high stakes faculty development project. Attending physicians on our ED call schedule were required to participate in order to remain on the ED admitting schedule. Whereas several attendings rated their experience during this faculty development study as positive, most of the attendings who participated in this faculty development project were community-based, IM practitioners with time constraints and did not view themselves as educators. The intensive resource investment required for this OSTE must be considered, given that all clinicians involved in a resident training program may not be fully invested in improving their communication and teaching skills. Because all academic medical school faculty are expected to provide a robust educational experience for residents in training, it may be appropriate to identify a group of self-motivated and interested attendings that could be targeted for regular and ongoing intervention [12].

The outcomes of the intervention demonstrate a positive trend supporting the implementation of this faculty development initiative. Attending physician teaching/feedback and communication skills, HCAHPS scores and IMR evaluations of attendings improved after the OSTE. We used a novel approach to faculty development with attendings as ‘learners’ and MS4s as ‘interns.’ Furthermore, our results comparing pre- and post-OSTE data sets represent an important contribution to the literature. We were able to take the attending experience in a standardized setting and apply it to the actual hospital setting where attendings interact with patients and residents. Regular intervention for selected faculty utilizing this unique OSTE format may provide a sustained benefit for enhancing communication and teaching skills, patient satisfaction, and overall resident training.
